# Comparing recruitment, retention, and safety reporting among geographic regions in multinational Alzheimer’s disease clinical trials

**DOI:** 10.1186/s13195-015-0122-5

**Published:** 2015-06-25

**Authors:** Joshua D Grill, Rema Raman, Karin Ernstrom, Paul Aisen, Sherie A Dowsett, Yun-Fei Chen, Hong Liu-Seifert, Ann Marie Hake, David S Miller, Rachelle S Doody, David B Henley, Jeffrey L Cummings

**Affiliations:** Institute for Memory Impairments and Neurological Disorders Department of Psychiatry and Human Behavior 3206 Biological Sciences, University of California, Irvine, CA 92697-4545 USA; Alzheimer’s Disease Cooperative Study, University of California, San Diego, San Diego, CA USA; Eli Lilly & Company, Indianapolis, IN USA; Bracket, Wayne, PA USA; Department of Neurology, Baylor College of Medicine, Houston, TX USA; Cleveland Clinic Lou Ruvo Center for Brain Health, Las Vegas, NV USA

## Abstract

**Introduction:**

Most Alzheimer’s disease (AD) clinical trials enroll participants multinationally. Yet, few data exist to guide investigators and sponsors regarding the types of patients enrolled in these studies and whether participant characteristics vary by region.

**Methods:**

We used data derived from four multinational phase III trials in mild to moderate AD to examine whether regional differences exist with regard to participant demographics, safety reporting, and baseline scores on the Mini Mental State Examination (MMSE), the 11-item Alzheimer’s Disease Assessment Scale–Cognitive subscale (ADAS-cog11), the Clinical Dementia Rating scale Sum of Boxes (CDR-SB), the Alzheimer’s Disease Cooperative Study–Activities of Daily Living Inventory (ADCS-ADL), and the Neuropsychiatric Inventory (NPI). We assigned 31 participating nations to 7 geographic regions: North America, South America/Mexico, Western Europe/Israel, Eastern Europe/Russia, Australia/South Africa, Asia, and Japan.

**Results:**

North America, Western Europe/Israel, and Australia/South Africa enrolled similar proportions of men, apolipoprotein E ε4 carriers, and participants with spouse study partners, whereas Asia, Eastern Europe/Russia, and South America/Mexico had lower proportions for these variables. North America and South America/Mexico enrolled older subjects, whereas Asia and South America/Mexico enrolled less-educated participants than the remaining regions. Approved AD therapy use differed among regions (range: 73% to 92%) and was highest in North America, Western Europe/Israel, and Japan. Dual therapy was most frequent in North America (48%). On the MMSE, North America, Western Europe/Israel, Japan, and Australia/South Africa had higher (better) scores, and Asia, South America/Mexico, and Eastern Europe/Russia had lower scores. Eastern Europe/Russia had more impaired ADAS-cog11 scores than all other regions. Eastern Europe/Russia and South America/Mexico had more impaired scores for the ADCS-ADL and the CDR-SB. Mean scores for the CDR-SB in Asia were milder than all regions except Japan. NPI scores were lower in Asia and Japan than in all other regions. Participants in North America and Western Europe/Israel reported more adverse events than those in Eastern Europe/Russia and Japan.

**Conclusions:**

These findings suggest that trial populations differ across geographic regions on most baseline characteristics and that multinational enrollment is associated with sample heterogeneity. The data provide initial guidance with regard to the regional differences that contribute to this heterogeneity and are important to consider when planning global trials.

## Introduction

Alzheimer’s disease (AD) is a worldwide pandemic. Between 1990 and 2010, the global health care burden caused by AD increased 244% [1]. The rapid increases in prevalence and cost have led several countries to develop national plans to address AD [2]. A goal of these plans is to advance research toward improved therapies and, in particular, drugs capable of slowing the course of the disease and delaying its onset if their use is initiated early enough. Key to developing improved AD therapies will be the conduct of robust clinical trials. AD trials present many challenges, including slow recruitment.

Most AD trials are now multinational [3,4]. Multinational trials enable expedited recruitment and are necessary to secure multinational regulatory registration and eventual patient access [5]. Yet, these trials may also bring ethical, logistical, and scientific challenges. Trials are usually conducted only in regions in which the drug, if approved, is available [6]. Some countries have instituted laws intended to protect citizens that may impede research conduct, and sponsors must negotiate local regulatory issues [7]. Translated study materials may introduce instructional and cultural inaccuracies, resulting in excess psychometric variance and reduced data integrity [8]. Global and ethnic variation in drug pharmacokinetics or pharmacodynamics may impact drug safety or efficacy [9,10].

For AD trials specifically, local laws, ethical guidelines, or practices regarding surrogate consent may vary among geographic regions [11,12]. Regional or cultural differences may affect whether and when a diagnosis is made, who provides care, and the availability of approved therapeutic options [13]. These and other factors could introduce heterogeneity into AD trial samples and should be considered when implementing multinational trials.

Despite the widespread dependence on multinational trials, there is little in the way of a “science of globalization” to inform decisions. To help address this information gap, we examined the baseline characteristics of trial participants across seven geographic regions in four multinational, phase III, industry-sponsored trials with patients with mild to moderate AD. We examined demographic as well as disease- and trial-related variables across geographic regions and compared regions for differences in the frequency of reported adverse events and participant study completion. For all outcomes, we tested the null hypothesis that geographic regions do not differ from each other in the setting of multinational AD trials. These exploratory analyses were conducted with the intention of generating data-based observations of participant characteristics and safety reporting across regions that may be helpful in trial planning. Measures of disease progression and the implications of these observations for trial planning and policy are reported separately.

## Methods

### Data source

These results describe a combined dataset from four multinational, phase III clinical trials conducted in mild to moderate AD. The results of the primary efficacy analyses from these trials have been reported elsewhere [14,15]. We analyzed data from two trials each of two investigational compounds, the γ-secretase inhibitor semagacestat [16-20] (the IDENTITY program: ClinicalTrials.gov identifiers NCT00762411 and NCT01035138) and the humanized monoclonal anti-amyloid-β (anti-Aβ) antibody solanezumab [21,22] (the EXPEDITION program: ClinicalTrials.gov identifiers NCT00905372 and NCT00904683). Each trial was sponsored by Eli Lilly & Company, and data were analyzed by the Alzheimer’s Disease Cooperative Study (ADCS) group members through its Data Analysis and Publication Committee. For each analysis, all available data were used.

### Trial inclusion and exclusion criteria

The four trials used nearly identical inclusion and exclusion criteria, though they varied according to the type of therapy under investigation. The semagacestat trials required the ability to swallow oral medications, and the solanezumab trials required good venous access for delivery of intravenous therapy and excluded those with allergies to humanized monoclonal antibodies. The solanezumab, but not semagacestat, trials excluded patients with a history of repeated head trauma over the previous 5 years.

Participants were at least 55 years of age and met National Institute of Neurological and Communicative Disorders and Stroke-Alzheimer’s Disease and Related Disorders Association criteria for probable AD [23]. Mild to moderate AD was defined as a score of 16 to 26 (inclusive) on the Mini Mental State Examination (MMSE) [24]. Participants were permitted to receive background cholinesterase inhibitors and/or memantine if the treatment was initiated at least 4 months prior to screening and was stable in dose for at least 2 months. They had to have had magnetic resonance imaging (MRI) or computed tomography (CT) results within the previous 2 years that were not inconsistent with a diagnosis of AD. Those without imaging had MRI and/or CT at screening.

All participants had a reliable caregiver who was in frequent contact with them (defined as ≥10 hours per week), accompanied them to site visits or was available by telephone, and monitored administration of prescription medications during the trial.

Participants were excluded if they had a Geriatric Depression Scale score >6, if they had a Hachinski Ischemic Score >4, or if they met the National Institute of Neurological Disorders and Stroke/Association Internationale pour la Recherche et l’Enseignement en Neurosciences criteria for vascular dementia [25]. Patients with serious or unstable medical conditions (including HIV) or a history within the last 5 years of serious central nervous system infection, primary or recurrent malignant disease (with the exception of resected cutaneous *in situ* squamous or basal cell carcinoma or *in situ* cervical or prostate cancer with normal prostate-specific antigen posttreatment), or chronic alcohol or drug abuse were excluded. Previous exposure to either the agent under study or an Aβ vaccine or monoclonal antibody was not permitted.

### Outcome measures

We examined the effect of geographic region on screening and baseline clinical outcome measures that are common to AD trials. A centralized company translated outcome measures into the appropriate language of the region of each site.

The MMSE is a global cognition measure that requires approximately 10 minutes to administer and is the most common tool for determining trial eligibility. Its items are used to assess short-term memory, orientation, calculation, language interpretation, naming, and praxis. The MMSE has a range of 0 to 30, with higher scores representing better performance. We investigated MMSE scores at screening and baseline.

The Alzheimer’s Disease Assessment Scale–Cognitive subscale (ADAS-cog) is the only cognitive outcome measure that has been used to successfully demonstrate drug efficacy in mild to moderate AD registration trials. It was one of the co-primary outcomes for each of the four trials included in our present analysis. The ADAS-cog typically includes 11 subtests that assess the patient’s memory, orientation, comprehension, naming, word finding, and ideational and constructional praxis [26]. The range is 0 to 70, with higher scores representing greater cognitive impairment. We assessed baseline scores on the 11-item ADAS-cog (ADAS-cog11).

The Alzheimer’s Disease Cooperative Study Activities of Daily Living Inventory (ADCS-ADL) was the other co-primary outcome measure for the four trials. The scale is informant-based and is used to assess basic and instrumental activities of daily living. Scores range from 0 to 78, with higher scores representing greater functional independence [27]. We examined ADCS-ADL scores at baseline.

The Clinical Dementia Rating scale Sum of Boxes (CDR-SB) was a secondary outcome measure in each trial. The CDR is a global instrument that includes separate interviews of the patient and the informant. The investigator uses the interviews to assign severity scores (0, not demented; 0.5, questionable dementia; 1.0, mild dementia; 2.0, moderate dementia; or 3.0, severe dementia) for each of six “boxes,” including memory, orientation, judgment and problem solving, community affairs, home and hobbies, and self-care [28]. We examined the Sum of Boxes scores at baseline.

The Neuropsychiatric Inventory (NPI) is the most widely used scale for examining behavioral symptoms in the setting of AD trials. The study partner is asked to report the frequency and severity of 12 behavioral symptoms observed over the previous 4 weeks [29,30]. Each domain is assessed as present or absent. If present, the severity (1 to 3 points) and frequency (1 to 4 points) are scored. The severity and frequency are multiplied, and the scores across domains are summed for a total range of 0 to 144, with higher scores representing greater behavioral symptoms.

Raters who failed to meet minimum experience requirements for the outcome measures were required to participate in an enriched training program, including additional online and live training. All raters underwent live training on outcome measures at the principal investigator’s meeting and were required to pass qualification assessments on the co-primary outcome scales. As part of an in-study rating review program, screening MMSE in both study programs and ADAS-cog at baseline and 12 weeks (EXPEDITION program) or 52 weeks (IDENTITY program) were reviewed for scoring errors; raters underwent remedial training when indicated; and errors were subsequently corrected.

### Data analyses

Patients were enrolled in 31 different countries. Investigative sites were chosen after a careful feasibility assessment of experience in caring for patients with AD, experience in running AD trials, and experience of raters in administering the trial outcome measures. On the basis of the country of enrollment, participants were categorized into one of seven geographic regions: North America (United States and Canada), South America/Mexico (Argentina, Brazil, Chile, and Mexico), Western Europe/Israel (Belgium, Denmark, Finland, France, Germany, Israel, Italy, Spain, Sweden, and United Kingdom), Eastern Europe/Russia (Bulgaria, Hungary, Poland, Romania, Russia, Serbia, Turkey, and Ukraine), Australia/South Africa, Asia (China, India, Korea, and Taiwan), and Japan. We based our regional assignments on the work of Glickman and colleagues [5], who grouped patients in parts of the world with shared culture, history, geography, and linguistic features. Definitions were modified to allow combination of some countries that contained small samples due to participation in only one study program.

Data for drug and placebo-assigned participants from all four trials were included in the baseline data analyses (demographic summaries and screening and baseline scores on outcome measures). Mean age and level of education were quantified in years. We also examined the proportion of each region with varying levels of education: <8 years, 8 to 12 years, and >12 years. Mean height in centimeters and weight in kilograms were assessed, and body mass index (BMI; weight divided by height squared) was calculated for each participant. Participants who carried one or more copies of the ε4 allele of the apolipoprotein E (APOE) genotype were categorized as ε4 carriers. Participant study partners were categorized as spouse, adult child, or other at baseline.

Study retention and treatment-emergent adverse event (TEAE) and serious adverse event (SAE) reporting were examined separately by study program (IDENTITY or EXPEDITION) and by treatment group assignment (semagacestat, solanezumab, or placebo).

Study retention was defined as fulfilling all eligible visits. In the IDENTITY program, semagacestat dosing was halted prior to study completion. The studies were amended to follow study participants for 7 months after discontinuing semagacestat, but these data are not included in the present analyses. Because of this amendment, however, some participants are included as “completers” (that is, retained for all eligible visits), despite participating for less than the protocol-defined 18-month study period.

TEAEs were defined as adverse events that first occurred or worsened in severity compared with their maximum severity during the baseline period (between screening and baseline visits). We examined TEAE reporting in each study program for the placebo groups and for the higher-dose arms of each active drug (semagacestat 140 mg by mouth daily and solanezumab 400 mg intravenously every 4 weeks). To account for differences in time to site startup and differences in the time for trial conduct (the IDENTITY trials were amended to stop semagacestat prior to completion), TEAEs were reported as per patient per month. We also examined the proportion of TEAEs reported as SAEs among the regions.

Descriptive statistics are presented as mean ± standard deviation for continuous variables and count (%) for categorical variables, unless otherwise stated. For continuous baseline variables in which assumptions of normality were met, analysis of variance (ANOVA) and Levene’s test were used to examine the overall impact of geographic region. If the assumptions were not met, the Kruskal-Wallis test was performed. Categorical baseline variables, TEAE reporting, and study retention were compared across geographical regions using a χ^2^ test for independence. For variables in which an overall significant effect of region was present, pairwise comparisons between regions were performed using Tukey’s honestly significant difference (HSD) test (with the ANOVA), the Wilcoxon rank-sum test with the Holm’s adjustment for multiple comparisons (with the Kruskal-Wallis test), and χ^2^ test using the Holm’s adjustment for multiple comparisons (with the χ^2^ test).

We report significant differences if they reached a conservative significance level of *P* < 0.01. Statistical analysis was conducted using R version 2.14.0 statistical software [31].

### Ethics

For each trial, informed consent was provided by the participant or a legally authorized representative, in accordance with local regulations, and only after approval by the site’s institutional review board of record. The present study analyzing data collected across these clinical trials was reviewed by the University of California, Los Angeles Medical Institutional Review Board 3 and was deemed as not meeting the definition of human subjects research.

## Results

### Demographics of participants

In total, data from 4,694 participants were included in these analyses. Forty percent of all participants were enrolled in North America. The next highest enrolling region was Western Europe/Israel, with 981 participants (21%) enrolled. No other region enrolled more than 10% of the overall sample across trials (Table 1). We observed regional differences for each demographic variable examined (age: *P* < 0.0001 by ANOVA; weight: *P* < 0.001 by Kruskal-Wallis test; height: *P* < 0.001 by Kruskal-Wallis test; body mass index: *P* < 0.001 by Kruskal-Wallis test; sex: *P* < 0.001 by χ^2^ test; education: *P* < 0.001 by Kruskal-Wallis test; APOE genotype: *P* < 0.001 by χ^2^ test; study partner type: *P* < 0.001 by χ^2^ test).

**Table 1:** **Regional demographic and disease-related summaries of the participants at baseline** ^**a**^

	**North America**	**Western Europe**	**Australia/South Africa**	**Japan**	**Asia**	**Eastern Europe/Russia**	**South America/Mexico**	**Total sample**
N (%)	1,884 (40.1)	981 (20.9)	237 (5.1)	435 (9.3)	339 (7.2)	408 (8.7)	410 (8.7)	4,694 (100)
Age, yr (mean ± SD)	75.1 ± 8.3^EE,AS,JP,AU,WE^	71.9 ± 7.8^SA,JP^	72.9 ± 7.4^SA,EE,NA^	73.4 ± 7.6^SA,NA^	72.1 ± 7.6^SA,NA^	70.7 ± 7.8^SA,AU,NA^	75.4 ± 7.7^EE,AS,JP,AU,WE^	73.6 ± 8.1
Female sex, n (%)	986 (52.3)^SA,EE,JP^	503 (51.3) ^SA,EE,JP^	128 (54.0)^SA^	276 (63.5)^WE,NA^	191 (56.3)	255 (62.5)^WE,NA^	279 (68.1)^AU,WE,NA^	2,618 (55.8)
Height, cm (mean ± SD)	166.5 ± 10.7^SA,EE,AS,JP^	166.1 ± 9.8^SA,EE,AS,JP^	166.8 ± 9.7^SA,EE,AS,JP^	154.6 ± 8.9^SA,EE,AS,AU,WE,NA^	158.2 ± 8.6^EE,JP,AU,WE,NA^	163.4 ± 9.1^SA,AS,JP,AU,WE,NA^	160.0 ± 9.1^EE,JP,AU,WE,NA^	163.9 ± 10.7
Weight, kg (mean ± SD)	73.2 ± 15.7^SA,EE,AS,JP,WE^	70.2 ± 12.7^SA,AS,JP,NA^	70.7 ± 13.1^SA,AS,JP^	53.1 ± 10.0^SA,EE,AS,AU,WE,NA^	58.5 ± 9.7^SA,EE,JP,AU,WE,NA^	68.8 ± 12.6^AS,JP,NA^	66.6 ± 12.5^AS,JP,AU,WE,NA^	68.6 ± 15.0
Body mass index, kg/m^2^ (mean ± SD)	26.3 ± 4.7^AS,JP,WE^	25.4 ± 3.8^AS,JP,NA^	25.4 ± 4.1^AS,JP^	22.1 ± 3.1^SA,EE,AS,AU,WE,NA^	23.3 ± 3.1^SA,EE,JP,AU,WE,NA^	25.4 ± 3.8^AS,JP^	26.0 ± 4.3^AS,JP^	25.4 ± 4.4
Years of education (mean ± SD)	14.1 ± 3.3^SA,EE,AS,JP,AU,WE^	11.2 ± 4.2^SA,EE,AS,NA^	12.1 ± 3.5^SA,AS,WE,NA^	11.7 ± 2.7^SA,AS,NA^	9.5 ± 4.7^EE,JP,AU,WE,NA^	11.9 ± 3.8^SA,AS,WE,NA^	8.9 ± 4.5^EE,JP,AU,WE,NA^	12.2 ± 4.1
APOE ε4 genotype carriers, n (%)	1,086 (63.2)^SA,EE,AS,JP^	494 (63.3)^SA,EE,AS,JP^	149 (63.7)^SA^	220 (51.9)^WE,NA^	93 (48.4)^WE,NA^	191 (51.1)^WE,NA^	189 (49.5)^AU,WE,NA^	2,422 (59.0)
Years since symptom onset (mean ± SD)	4.8 ± 2.6^EE,AS,JP^	4.6 ± 2.5^EE,JP^	4.7 ± 2.8^EE,JP^	3.7 ± 2.3^SA,AS,AU,WE,NA^	4.2 ± 2.4^JP,NA^	3.9 ± 2.2^SA,AU,WE,NA^	4.5 ± 2.4^EE,JP^	4.5 ± 2.5
Years since diagnosis (mean ± SD)	2.5 ± 2.1^EE,AS,JP,AU,WE^	2.1 ± 1.8^EE,JP,NA^	2.0 ± 1.8^EE,NA^	1.7 ± 1.5^SA,WE,NA^	2.0 ± 1.9^SA,EE,NA^	1.5 ± 1.5^SA,AS,AU,WE,NA^	2.4 ± 1.9^EE,AS,JP^	2.2 ± 1.9
Proportion taking any anti-AD medication, n (%)	1,677 (89.0) ^EE,AS,AU^	902 (92.0)^SA,EE,AS,AU^	172 (72.6)^JP,WE,NA^	389 (89.4)^AS,AU^	274 (80.8)^WE,NA^	302 (74.0)^JP,WE,NA^	342 (83.4)^WE^	4,058 (86.5)
Proportion taking dual anti-AD therapy, n (%)	894 (47.5)^SA,EE,AS,JP,AU,WE^	147 (15.0)^SA,AS,JP,AU,NA^	16 (6.8)^SA,JP,WE,NA^	3 (0.7)^SA,EE,AS,AU,WE,NA^	21 (6.2)^SA,EE,JP,WE,NA^	58 (14.2)^SA,AS,JP,NA^	117 (28.5)^EE,AS,JP,AU,WE,NA^	1,256 (26.8)
Proportion enrolling with spouse study partner, n (%)	1,318 (70.4)^SA,EE,AS^	719 (73.7)^SA,EE,AS^	178 (75.7)^SA,EE,AS^	279 (64.6)^SA,EE,AS^	172 (50.9)^JP,AU,WE,NA^	163 (40.1)^JP,AU,WE,NA^	174 (42.7)^JP,AU,WE,NA^	3,003 (64.4)
Proportion enrolling with adult child study partner, n (%)	374 (20.0)	192 (19.7)	36 (15.3)	116 (26.9)	136 (40.2)	203 (49.9)	162 (39.7)	1,219 (26.1)
Proportion enrolling with other study partner, n (%)	179 (9.6)	64 (6.6)	21 (8.9)	37 (8.6)	30 (8.9)	41 (10.1)	72 (17.7)	444 (9.5)

^a^SA = *P* < 0.01 vs South America/Mexico; EE = *P* < 0.01 vs Eastern Europe/Russia; AS = *P* < 0.01 vs Asia; JP = *P* < 0.01 vs Japan; AU = *P* < 0.01 vs Australia/South Africa; WE = *P* < 0.01 vs Western Europe; NA = *P* < 0.01 vs North America; AD = Alzheimer’s disease; APOE = Apolipoprotein E; Dual therapy = treatment with a cholinesterase inhibitor and memantine; SD = Standard deviation.

In pairwise comparisons, participants enrolled in North America and South America/Mexico were older than those enrolled in every other region (Table 1). Participants enrolled in Eastern Europe/Russia were the youngest (*P* < 0.001 for all comparisons except vs Australia/South Africa (*P* = 0.011), Western Europe/Israel (*P* = 0.09), and Asia (*P* = 0.20), all by Tukey’s HSD test).

North American participants were taller than participants from every other region (*P* < 0.001 by paired Wilcoxon rank-sum test with Holm’s adjustment) except Australia/South Africa and Western Europe/Israel and heavier than participants from every other region (*P* < 0.001) except Australia/South Africa (Table 1). Japanese participants were lighter, shorter, and had lower BMIs than participants from every other region (*P* < 0.001). Excluding Japan, Asian participants were lighter, shorter, and had lower BMIs than those in the remaining regions (*P* < 0.001 for all comparisons except vs South America/Mexico).

In every region, more women than men were enrolled. In South America/Mexico, 68% of participants were female, the highest proportion of any region (*P* < 0.01 vs Australia/South Africa, North America, and Western Europe/Israel and *P* = 0.02 vs Asia, both by χ^2^ test with Holm’s adjustment). Western Europe/Israel, North America, and Australia/South Africa enrolled the highest proportions of male participants.

The range of education among participants was 0 to 29 years, with an overall median education level of 12 years for the combined dataset. Participants from North America had higher education than participants from all other regions, with 60% of participants having >12 years and <3% of participants having <8 years (data not shown). Japan had a similar low proportion of participants with <8 years of education (2.8%), but the majority of Japanese participants (74.7%) had <12 years. Participants from South America/Mexico (mean = 8.9 years) and Asia (mean = 9.5 years) had less education than all other regions (Table 1). These regions had substantially higher proportions of participants with <8 years of education (39% for Asia and 49% for South America/Mexico; data not shown) than all other regions.

Fifty-nine percent of all participants carried at least one copy of APOE ε4. The proportions of APOE ε4 carriers ranged from 48.4% (Asia) to 63.7% (Australia/South Africa). North America, Western Europe/Israel, and Australia/South Africa had the highest proportions of APOE ε4 carriers (*P* < 0.01 by χ^2^ test with Holm’s adjustment for all comparisons to North America and Western Europe/Israel) (Table 1).

In North America, Western Europe/Israel, and Australia/South Africa, >70% of participants were enrolled with a spouse study partner. In contrast, the majority of participants in Eastern Europe/Russia (60%) and South America/Mexico (57%) were enrolled with a nonspouse study partner. In Eastern Europe/Russia, 50% of participants were enrolled with an adult child, and in South America/Mexico, 18% of participants were enrolled with a study partner who was neither a spouse nor an adult child—higher proportions, respectively, than any other region.

### Disease-related variables

Geographic regions differed in the time since symptom onset and time between diagnosis and trial enrollment (*P* < 0.001 for both variables by Kruskal-Wallis test). The overall mean duration of symptoms prior to enrollment was 4.5 ± 2.5 years. This duration was significantly shorter in Japan and Eastern Europe/Russia than all other regions except Asia (*P* < 0.01 for all comparisons except Eastern Europe/Russia vs Asia) (Table 1). North America had the longest duration of symptoms prior to enrollment, though the difference reached statistical significance only when compared with Japan, Eastern Europe/Russia, and Asia. The mean time from diagnosis to enrollment was approximately 2.0 to 2.5 years shorter than the time since symptom onset for each region, with a pattern of pairwise differences similar to that observed for time since symptom onset. Eastern Europe/Russia and Japan had the shortest duration of time since diagnosis (*P* < 0.01 for all comparisons except Japan vs Asia) (Table 1). North America had longer duration of time since diagnosis than all other regions except South America/Mexico.

Across geographic regions, a large majority (86.5%) of participants were taking at least one US Food and Drug Administration–approved anti-AD medication. Among AD medications, donepezil was most common; 52% of all participants were taking donepezil at the time of screening. Anti-AD drug use at screening varied significantly among geographic regions, however (χ^2^; *P* = 0.0001 by χ^2^ test). Anti-AD drug use was highest in Western Europe/Israel, North America, and Japan (*P* < 0.01 for comparisons to remaining regions except North America vs South America/Mexico (*P* = 0.019) and Japan vs Asia (*P* = 0.012)). Memantine use was less common than cholinesterase inhibitor therapy; 32% of participants were taking memantine and 27% were on dual therapy at the time of screening. Both memantine and dual therapy rates differed among the regions (*P* < 0.0001 by χ^2^ test). More participants in North America than in any other region were on dual therapy. Fewer participants in Japan than in any other region were on dual therapy.

### Baseline outcome measure scores

Scores on cognitive, functional, and behavioral outcomes at screening and baseline visits differed among the regions (*P* < 0.0001 for each outcome measure by Kruskal-Wallis test). Despite the study inclusion criteria (MMSE score between 16 and 26), the range of MMSE scores observed at screening was 13 to 27. Only Japan and Western Europe/Israel did not enroll a participant with a screening MMSE score outside the inclusion criteria, though no region exceeded 1% of scores out of range at screening. Higher mean MMSE scores at screening were observed in North America, Western Europe/Israel, Australia/South Africa, and Japan relative to the remaining regions (Table 2). The mean MMSE scores and patterns of regional differences at baseline remained largely the same as at screening, but the variance increased in each region at baseline (Table 2). The range of MMSE scores at the baseline visit was from 6 to 30. Overall, 7.5% of all baseline visit scores were outside the screening range of 16 to 26. Eleven percent of baseline visit MMSE scores in North America and 9.5% in Asia were outside the screening entry criteria.

**Table 2:** **Baseline scores across regions** ^**a**^

	**North America**	**Western Europe**	**Australia/South Africa**	**Japan**	**Asia**	**Eastern Europe/Russia**	**South America/Mexico**	**Total sample**
MMSE at screen, mean ± SD	21.0 ± 3.2^SA,EE,AS^	21.0 ± 3.1^SA,EE,AS^	21.0 ± 3.0^SA,EE,AS^	20.9 ± 2.9^SA,EE,AS^	19.8 ± 3.1^JP,AU,WE,NA^	20.1 ± 3.0^JP,AU,WE,NA^	20.2 ± 3.0^JP,AU,WE,NA^	20.8 ± 3.1
MMSE at baseline, mean ± SD	21.0 ± 3.7^SA,EE,AS^	20.9 ± 3.4^SA,EE,AS^	20.8 ± 3.4^AS^	20.9 ± 3.3^AS^	19.8 ± 3.4^JP,AU,WE,NA^	20.3 ± 3.1^WE,NA^	20.3 ± 3.1^WE,NA^	20.7 ± 3.5
ADAS-cog11, mean ± SD	22.0 ± 8.9^SA,EE,AS,WE^	23.4 ± 8.9^SA,EE,JP,NA^	21.9 ± 9.2^SA,EE,AS^	21.4 ± 6.7^SA,EE,AS,WE^	24.1 ± 7.9^EE,JP,AU,NA^	27.3 ± 10.7^AS,JP,AU, WE,NA^	25.0 ± 8.8^JP,AU,WE,NA^	23.1 ± 9.0
ADCS-ADL, mean ± SD	62.4 ± 11.7^SA,EE,AS,JP,WE^	59.3 ± 13.3^SA,EE,NA^	59.6 ± 13.3^SA,EE^	60.2 ± 11.4^SA,EE,NA^	56.5 ± 14.5^SA,EE,NA^	50.9 ± 15.7^AS,JP, AU, WE,NA^	53.3 ± 14.4^AS,JP,AU, WE,NA^	59.2 ± 13.5
CDR-SB, mean ± SD	5.1 ± 2.5^SA,EE,AS^	5.3 ± 2.6^SA,EE,AS^	5.5 ± 2.6^SA,EE,AS^	5.1 ± 2.8^SA,EE^	4.7 ± 2.6^SA,EE,AU,WE,NA^	7.0 ± 3.2^AS,JP,AU, WE,NA^	6.4 ± 2.9^AS,JP,AU,WE,NA^	5.4 ± 2.7
NPI, mean ± SD	9.2 ± 10.9^SA,JP,AU, WE^	10.5 ± 11.3^AS,JP,NA^	11.9 ± 12.1^AS,JP,NA^	6.6 ± 8.6^SAEE,AU,WE,NA^	7.7 ± 9.2^SA,EE,AU,WE^	10.7 ± 11.6^AS,JP^	11.9 ± 12.1^AS,JP,NA^	9.6 ± 11.0

^a^SA = *P* < 0.01 vs South America/Mexico; EE = *P* < 0.01 vs Eastern Europe/Russia; AS = *P* < 0.01 vs Asia; JP = *P* < 0.01 vs Japan; AU = *P* < 0.01 vs Australia/South Africa; WE = *P* < 0.01 vs Western Europe; NA = *P* < 0.01 vs North America; ADAS-cog11 = 11-item Alzheimer’s Disease Assessment Scale–Cognitive subscale;; ADCS-ADL = Alzheimer’s Disease Cooperative Study–Activities of Daily Living Inventory; CDR-SB = Clinical Dementia Rating scale Sum of Boxes; MMSE = Mini Mental State Examination.

Baseline scores on the ADAS-cog11 ranged from 3 to 68. Mean scores in North America, Australia/South Africa, and Japan were significantly milder than those for all remaining regions (*P* < 0.01 for all comparisons except Australia/South Africa vs Western Europe/Israel (*P* = 0.09)). Eastern Europe/Russia demonstrated significantly higher scores than all remaining regions (*P* < 0.01 for all comparisons except vs South America/Mexico (*P* = 0.03)).

Participants from Eastern Europe/Russia and South America/Mexico performed worse (greater disease severity) than those from all other regions for both the ADCS-ADL and the CDR-SB (*P* < 0.01 for all comparisons by Wilcoxon rank-sum test). ADCS-ADL scores in North America were higher (less functional impairment) than in all other regions (*P* < 0.01 by Wilcoxon rank-sum test for all comparisons except Australia/South Africa (*P* = 0.015)). Mean CDR-SB scores in Asia were milder than in all regions except Japan (*P* < 0.01 for all comparisons by Wilcoxon rank-sum test).

Australia/South Africa and South America/Mexico had the highest NPI scores at baseline (greater neuropsychiatric symptomatology). Japan had significantly lower scores than all other regions except Asia (*P* < 0.01 for all comparisons by Wilcoxon Rank Sum test) (Table 2).

### Treatment-emergent adverse event reporting

The overall reporting of TEAEs for the four examined datasets was 77% for the IDENTITY program placebo arms, 89% for the IDENTITY semagacestat arms, 84% for the EXPEDITION placebo arms, and 81% for the EXPEDITION solanezumab arms. TEAE reporting among regions ranged from 57% for Eastern Europe/Russia in the IDENTITY program placebo arms to 95% for North America in the IDENTITY 140-mg dose semagacestat arms. TEAE reporting normalized by time and participant differed among regions for each dataset (*P* < 0.0001 for all by χ^2^ test), and the observed geographic patterns were similar for both agents and both placebo datasets. North America and Western Europe/Israel performed similarly and had significantly more reported TEAEs than Eastern Europe/Russia and Japan in most datasets (Table 3). Asia and Eastern Europe/Russia performed similarly in most analyses and had fewer TEAEs. There were no differences between regions in TEAEs severe enough to lead to discontinuation (Table 4).

**Table 3:** **Treatment-emergent adverse event rates per participant per month for combined trial arms among regions** ^**a**^

**Study program**	**Treatment group**	**North America**	**Western Europe**	**Australia/South Africa**	**Japan**	**Asia**	**Eastern Europe/Russia**	**South America/Mexico**
IDENTITY	Placebo	0.52 ± 1.31^SA,EE,JP^	0.37 ± 0.78^EE,JP^	0.34 ± 0.48	0.14 ± 0.17 ^WE,NA^	0.31 ± 0.63	0.31 ± 0.84^WE,NA^	0.25 ± 0.56^NA^
	Semagacestat 140 mg PO	1.10 ± 1.59^EE,JP^	0.76 ± 1.02^EE^	0.87 ± 0.85^EE^	0.57 ± 0.86^NA^	0.69 ± 0.89	0.62 ± 1.28^AU,WE,NA^	0.88 ± 1.36
EXPEDITION	Placebo	0.40 ± 0.57^SA,EE,JP,WE^	0.24 ± 0.27^SA,EE,NA^	0.42 ± 0.31^SA,EE, JP,WE^	0.16 ± 0.13^AU,NA^	0.31 ± 0.42^EE^	0.19 ± 0.40^AS,AU,WE,NA^	0.22 ± 0.28^AU,NA^
	Solanezumab 400 mg IV	0.41 ± 0.68^SA,EE,JP,WE^	0.24 ± 0.63^NA^	0.51 ± 1.04^SA,EE^	0.21 ± 0.22^NA^	0.29 ± 0.68^EE^	0.11 ± 0.13^AS,AU,NA^	0.19 ± 0.29^AU,NA^

^a^Data are presented as mean ± standard deviation. SA = *P* < 0.01 vs South America/Mexico; EE = *P* < 0.01 vs Eastern Europe/Russia; AS = *P* < 0.01 vs Asia; JP = *P* < 0.01 vs Japan; AU = *P* < 0.01 vs Australia/South Africa; WE = *P* < 0.01 vs Western Europe; NA = *P* < 0.01 vs North America; IV = Intravenously; PO = By mouth.

**Table 4:** **Most frequent reasons for drop out across global regions** ^**a**^

**Study program**	**Treatment group**	**North America**	**Western Europe**	**Australia/South Africa**	**Japan**	**Asia**	**Eastern Europe/Russia**	**South America/Mexico**
IDENTITY	Placebo	1. AEs = 43 (48%)	1. AEs = 19 (51%)	1. CG decision = 4 (44%)	1. AEs = 4 (36%)	1. Participant decision = 9 (45%)	1. Participant decision = 32 (59%)	1. Participant decision = 6 (38%)
		2. Participant decision = 15 (17%)	2. Participant decision = 9 (24%)	1. Participant decision = 4 (44%)	2. CG decision = 3 (27%)	2. AEs = 6 (30%)	2. AEs = 15 (28%)	2. CG decision = 3 (19%)
					2. Death = 3 (27%)			2. AEs = 3 (19%)
	Semagacestat 140 mg PO	1. AEs = 105 (55%)	1. AEs = 55 (66%)	1. AEs = 12 (48%)	1. AEs = 28 (74%)	1. AEs = 18 (47%)	1. AEs = 33 (46%)	1. AEs = 25 (49%)
		2. Participant decision = 38 (20%)	2. Participant decision = 12 (14%)	2. Death = 5 (20%)	2. CG decision = 6 (16%)	2. Participant decision = 13 (34%)	2. Participant decision = 12 (42%)	2. Participant decision = 13 (25%)
EXPEDITION	Placebo	1. AEs = 39 (31%)	1. AEs = 19 (38%)	1. AEs = 3 (50%)	1. AEs = 5 (50%)	1. Participant decision = 4 (36%)	1. Participant decision = 9 (38%)	1. Participant decision = 10 (36%)
		2. CG decision = 33 (26%)	2. Participant decision = 14 (28%)	2. CG decision = 1 (17%)	2. Participant decision = 2 (20%)	2. AEs = 2 (18%)	2. AEs = 5 (21%)	2. AEs = 7 (25%)
				2. Physician decision = 1 (17%)		2. Death = 2 (18%)		
				2. Death = 1 (17%)				
	Solanezumab 400 mg IV	1. CG decision = 39 (27%)	1. AEs = 16 (38%)	1. AEs = 7 (64%)	1. AEs = 5 (45%)	1. AEs = 2 (25%)	1. CG decision = 6 (43%)	1. Participant decision = 6 (29%)
		2. AEs = 34 (24%)	2. Participant decision = 10 (24%)	2. Participant decision = 2 (18%)	2. CG decision = 4 (36%)	1. Participant decision = 2 (25%)	2. AEs = 3 (21%)	2. AEs = 5 (24%)
				2. Physician decision = 2 (18%)		1. Protocol violation = 2 (25%)	2. Participant decision = 3 (21%)	

^a^Data are presented as count (%). AE = Adverse event; CG = Caregiver; IV = Intravenously; PO = By mouth.

The overall reporting of SAEs was 12% for the IDENTITY program placebo arms, 21% for the IDENTITY semagacestat arms, 20% for the EXPEDITION placebo arms, and 18% for the EXPEDITION solanezumab arms. We found no regional differences in SAE reporting.

### Participant retention

**Figure 1:**
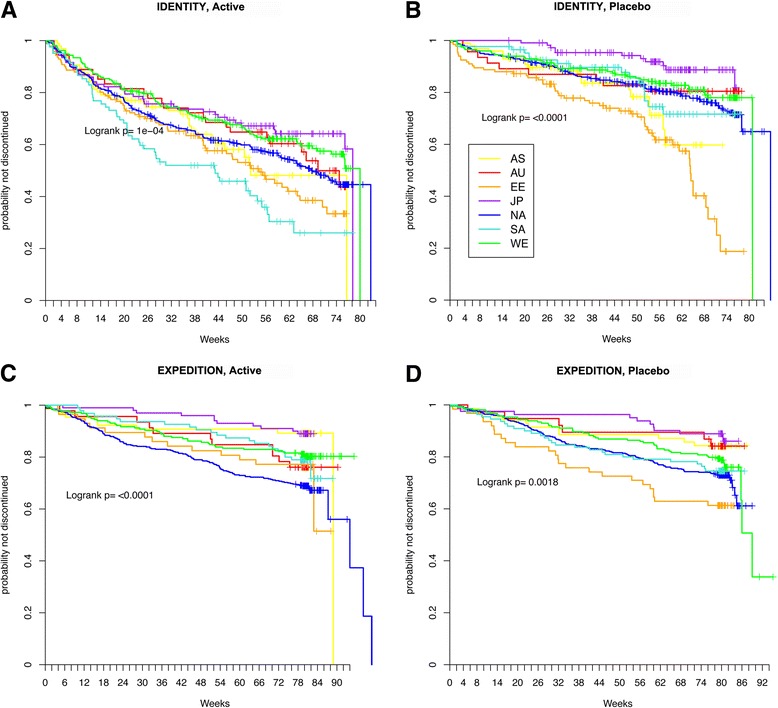
**Time to discontinuation models.** In time to early discontinuation models for each study program arm, Japan differed significantly from South America/Mexico in the IDENTITY active semagacestat arms **(A)**, from Asia and Eastern Europe/Russia in the IDENTITY placebo arms **(B)**, from North America in the EXPEDITION active solanezumab treatment arms **(C)**, and from Eastern Europe/Russia for the EXPEDITION placebo arms **(D)** (*P* < 0.01 for all comparisons by log-rank test). Eastern Europe/Russia differed from Australia/South Africa, North America, and Western Europe/Israel in the IDENTITY placebo arms (*P* < 0.01 by log-rank test). South America/Mexico differed from Western Europe/Israel in the IDENTITY active semagacestat arms (*P* < 0.01 by log-rank test).

The proportions of participants discontinuing prior to trial completion were similar for the solanezumab (24%) and placebo datasets (25%) in the EXPEDITION program. In the IDENTITY program, discontinuation was 22% for the combined placebo arms but 46% for the combined semagacestat arms. In each study program (IDENTITY and EXPEDITION), the global regions differed in participant retention (*P* < 0.01 for each dataset by χ^2^ test). For each study program, the dropout rate was lowest in Japan (Table 5). The dropout rate was highest in Eastern Europe/Russia for each placebo dataset (39% in EXPEDITION and 41% in IDENTITY) and the semagacestat treatment arms (51%). The dropout rate was highest in North America for the solanezumab active treatment arms (32%). Figure 1 illustrates the results of a time to discontinuation model, in which Japan differed from at least one other region in placebo and active treatment arms of each study program.

**Table 5:** **Completion rates for combined trial arms among regions** ^**a**^

**Study program**	**Treatment group**	**North America**	**Western Europe**	**Australia/South Africa**	**Japan**	**Asia**	**Eastern Europe/Russia**	**South America/Mexico**
IDENTITY	Placebo	291 (76.4)^EE^	162 (81.4)^EE^	37 (80.4)	99 (90.0)^EE^	79 (79.8)	79 (59.4)	70 (81.4)
	Semagacestat 140 mg PO	198 (50.8)	129 (60.9)	29 (53.7)	69 (64.5)	58 (60.4)	69 (48.9)	31 (37.8)
EXPEDITION	Placebo	325 (72.1)	163 (76.5)	32 (84.2)	71 (87.7)	59 (84.3)	38 (61.3)	82 (74.6)
	Solanezumab 400 mg IV	303 (67.8)^JP^	175 (80.7)	35 (76.1)	89 (89.0)	57 (87.7)	43 (75.4)	74 (77.9)

^a^Data are presented as count (%). EE = *P* < 0.01 vs Eastern Europe; JP = *P* < 0.01 vs Japan; IV = Intravenously; PO = By mouth.

Across study programs and trial arms, the regions appeared similar in the reasons for discontinuation. The most common reasons for discontinuation were adverse events, subject decision, and caregiver decision (Table 4). Adverse events were the most frequent cause of discontinuation and were consistently the most common cause of discontinuation for each region in the IDENTITY active treatment arms. In Eastern Europe/Russia and South America/Mexico, subject decision was a more common cause of discontinuation for the remaining study program arms (Table 4).

## Discussion

### Summary

These results suggest that—despite strict protocols, ample site training, and substantial trial monitoring—significant heterogeneity should be expected among AD trial populations across geographic regions. Furthermore, we observed patterns of regional similarities and differences for participant demographics, scores on trial outcome measures at screening and baseline visits, TEAE reporting, and study completion.

North America, Western Europe/Israel, and Australia/South Africa were similar in their proportions of female participants, carriers of the APOE ε4 genotype, and participants enrolled with a spouse study partner. Proportions different from this group but similar to each other were observed for Asia, Eastern Europe/Russia, and South America/Mexico for the same variables. Similar regional patterns were observed when we compared scores on trial outcomes at screening and baseline. Though consistent patterns were evident, they seemed dependent upon whether the outcome measure was based on informant report. Participants from North America, Western Europe/Israel, Japan, and Australia/South Africa had milder scores for study partner–independent measures (that is, MMSE at screening and baseline and the ADAS-cog11), whereas participants from Asia, South America/Mexico, and Eastern Europe/Russia had more moderate severity for these outcomes. Eastern Europe/Russia had the most severe scores for the CDR-SB; the mildest CDR-SB scores were observed in Asia. Scores on informant-independent outcomes were generally mildest in Australia/South Africa; this region had the most severe scores on the NPI. Asia and Japan, in contrast, demonstrated substantially lower NPI scores than the remaining regions. Japan also had the lowest frequency of reporting TEAEs for three of the four datasets; Eastern Europe/Russia had lower reporting frequency for the solanezumab arms of the EXPEDITION program. The highest TEAE reporting was in North America and Australia/South Africa.

### Potential explanations for the observed heterogeneity

We hypothesize that several factors that are not mutually exclusive contributed to the observed heterogeneity. First, the regions in which participants were recruited are different. Geographic regions differ in lifestyle factors, overall health, and causes of death and disability [32,33]. It is likely that access to medical care and the sophistication of that care differ among geographic regions. The populations recruited to these studies may accurately represent differences among the disease-suffering populations in different parts of the world. For example, North American participants had substantially higher levels of education than did those in South America/Mexico and Asia, as is the case for the countries in these regions [3]. It is important to note, however, that in North America—and probably every other region—trials are subject to sample bias. In the United States, trial populations are consistently more educated than the general population. Thus, these findings may reflect regional differences in population demographics as well as regional differences in the degree of sample bias; that is, patient access to trials and willingness to participate may differ among regions.

Regional differences in AD diagnosis, care, and reimbursement may also have contributed to the observed heterogeneity. Until recently, the only AD therapy that had received regulatory approval in Japan was donepezil [34]. This may explain or contribute to the low frequencies of memantine and dual therapy in Japan. Other regional differences in standard of care or physician reimbursements for diagnostic visits or procedures could similarly impact the stage of disease at which a formal diagnosis is made, and this could have an impact on variables such as time from symptom onset to trial screening and baseline disease severity. In fact, the regions with the shortest times from symptom onset and diagnosis to screening (Japan and Eastern Europe/Russia) did not have milder scores on baseline trial outcome measures than the regions with longer durations. Eastern Europe/Russia had the most severe scores at baseline. North America had the longest duration of symptoms and time since diagnosis to enrollment, but it had among the mildest scores on informant-independent baseline outcomes. Possible explanations for such discrepancies could be differing rates of disease progression among regions, differing access to medical care, or earlier detection in some regions, though this will require further study.

Regional variation in research infrastructure or the expertise of investigators could also have contributed to the observed heterogeneity. For example, the availability of experienced raters at sites varied across regions and such differences might impact mean scores or variability on trial outcomes at baseline. We cannot assume that differences in investigative teams explain the observed differences, however; it is possible that differences in patients, informants, outcomes (when translated, for example), and raters exist.

Translation of outcome measures does not guarantee equivalence among cultural groups or regions [35]. Local customs and standards may necessitate adjustment [36] or replacement [37] of particular items. For example, one Chinese version of the ADAS-cog used pictures instead of words for assessing memory performance [38]. Alternatively, findings from some studies suggest that differing cutoffs may be appropriate when applying common scales to differing geographic, ethnic, and cultural populations [39]. Even within geographic regions, as defined in the present study, challenges related to harmonization and validation of outcome measures may occur, potentially further increasing trial data variance [40]. In the studies examined here, scales were kept consistent to the greatest extent possible to facilitate combining study data; only in certain circumstances were sites permitted to alter scales for regional differences (for example, substituting region or burro for county, where counties were not present, on the MMSE).

Regional and cultural differences in family attitudes toward AD recognition, diagnosis, treatment, reporting of symptoms, and research participation may have contributed to the observed heterogeneity. In North America, Western Europe/Israel, and Australia/South Africa, patients with a spouse made up a majority of the participants and proportionately more men were enrolled. In contrast, the majority of participants in Eastern Europe/Russia and South America/Mexico enrolled with a nonspouse study partner. It is not clear whether regional differences exist in the proportions of caregiver types or if caregiver attitudes inhibit participation by nonspousal partners in some regions and enhance it in others. Cultural differences among caregivers may also have impacted informant reporting in the trials. TEAE reporting and scores on the CDR-SB and NPI were consistently lower for Asia and Japan, relative to the other regions, similar to previous observations [41].

Finally, regional ethnogenetic differences in disease may have contributed to the observed heterogeneity. This is most pertinent to the observed frequencies of APOE genotypes. The APOE ε4 genotype is the best replicated and most understood genetic risk factor for AD [42], but the impact of APOE (and other) genotypes on AD risk in different ethnic groups remains unclear [43]. APOE ε4 prevalence may differ regionally, possibly accounting for the difference in ε4 proportions observed in these analyses. For example, fewer participants carried APOE ε4 in Asia than in other regions, a finding similar to that of previous studies of APOE prevalence [44,45]. Alternatively, epigenetic differences may result in altered genetic risk for disease [46]. Here, APOE ε4 differences did not seem to predict differences in mean age between regions. North America had the highest rate of ε4 carriers and the oldest mean age, whereas Eastern Europe/Russia had the second-lowest proportion of ε4 carriers and a younger mean age than the other global regions. To the extent that drug interactions with genotype impact the safety [47] or efficacy of AD treatments [48-50], ethnogenetic differences within trial samples should be considered when implementing multinational trials. Differences in the proportions of ε4 carriers and noncarriers could also have specific implications for trials of antiamyloid therapies because noncarrier participants may more frequently fail to demonstrate amyloid burden when studied with amyloid imaging [51].

### Limitations

These data are among the first of their kind, and several limitations should be considered. Our observations do not provide evidence for why heterogeneity exists. Though we provide hypotheses related to factors that may contribute to the observed regional differences, these hypotheses require further research to better guide sponsors of multinational AD trials. Furthermore, because these study programs were not designed to evaluate regional differences, several data elements important to sponsors designing global trials were not sufficiently available to permit analysis, including regulatory startup variables such as time to institutional review board approval or contract negotiation, the type of sites and investigators within each global region, and participant data on socioeconomic status. The grouping of regions was based on geography, information in the published literature [5], and the experiences of the research team, and with data limitations in mind. Specifically, low numbers of participants in some countries or regions necessitated combinations to improve statistical power. This limitation may be minimized by the findings of significant regional differences. Were the data homogeneous, the assignment of regions, even if arbitrary, would not be expected to produce statistically significant differences among groups. The pattern of differences that we observed may not be the same in future datasets, however, so our results cannot be used to predict future findings in a specific region or country. Other strategies for assigning global regions, including ethnic or genetic groupings, might also be reasonable and could produce alternate findings.

Finally, although many of the differences between regions are statistically significant, it is unclear to what extent they are clinically meaningful or interfere with the ability to measure a drug effect. In the IDENTITY studies, for example, the cognitive worsening associated with semagacestat treatment was identified despite population heterogeneity.

### Impact

We performed these analyses to provide sponsors with data to assist with planning and conducting trials in multiple geographic regions. The data indicate that study populations differ across regions from a demographic perspective. Similarly, APOE ε4 carrier status differed among regions in these trials, and this may bear on the number of non-AD patients entering trials that do not utilize AD biomarkers as entry criteria. Screening and baseline scores on the outcome measures we examined differed among regions, again indicating the heterogeneity of multinational trial populations. The difference in TEAE reporting and dropout among regions is consistent with findings from a previous analysis by country of the IDENTITY trial data [52]. These data suggest that heterogeneity will be present and should be accounted for when developing multinational AD trials. Although researchers generally attempt to avoid heterogeneity in clinical trials to facilitate identifying a drug effect if one exists, heterogeneity may also provide confidence that an observed drug effect is real and that treatment will be effective in the general clinical population, where heterogeneity will be the norm.

## Conclusions

To meet regulatory and enrollment needs, sponsors of studies in AD and other serious diseases are increasingly implementing multinational clinical trials. Our data suggest that this may contribute to sample heterogeneity. Because trial designs and sample sizes are dependent upon expected population variance, these results suggest that (1) sponsors may wish to limit the number of regions from which sites outside the United States recruit participants to reduce variance, (2) multinational trials may need to be large enough to account for potentially increased variance, and (3) sponsors must carefully consider which countries and regions to include when planning multinational trials. For example, trials of interventions to reduce or prevent neuropsychiatric symptoms may face additional challenges in Japan and Asia, given lower reporting of these symptoms in those regions. Sponsors may also consider balancing enrollment sites, based on the knowledge of which regions are likely to enroll similar patients in terms of age, body size, genotypes, and concomitant therapies.

Although differences in the proportion of participants receiving anti-AD medications among the regions were evident, more than 70% of patients in each region were taking at least one anti-AD medication. This suggests that trial designs that seek to enroll drug-naïve participants will have increasingly challenging recruitment, even when enrolling non-US populations [4]. Moreover, to the extent that trial designs require patients to be on particular AD therapies, these data may instruct selection of regional sites.

To develop desperately needed new drugs for AD, high-quality clinical trials must be performed in a rapid manner. The conduct of multinational trials accelerates patient recruitment and enables broader registration and eventual patient access, but it introduces variables that have not been completely delineated and are incompletely understood. Trial sponsors must carefully consider potential effects on trial data and implement strategies to identify those factors that can be mitigated to reduce variability.

## Abbreviations

Aβ: Amyloid-β
AD: Alzheimer’s disease
ADAS-cog11: 11-item Alzheimer’s Disease Assessment Scale–Cognitive subscale
ADCS: Alzheimer’s Disease Cooperative Study
ADCS-ADL: Alzheimer’s Disease Cooperative Study–Activities of Daily Living Inventory
AE: Adverse event
ANOVA: Analysis of variance
APOE: Apolipoprotein E
BMI: Body mass index
CDR-SB: Clinical Dementia Rating scale Sum of Boxes
CG: Caregiver
CT: Computed tomography
HSD: Honestly significant difference
IV: Intravenous
MMSE: Mini Mental State Examination
MRI: Magnetic resonance imaging
NPI: Neuropsychiatric Inventory
PO: By mouth
SAE: Serious adverse event
SD: Standard deviation
TEAE: Treatment-emergent adverse event
